# Broadcasters’ Leadership Traits and Audiences’ Loyalty With the Moderating Role of Self-Construal: An Exploratory Study

**DOI:** 10.3389/fpsyg.2021.605784

**Published:** 2021-04-22

**Authors:** Yidan Huang, Yi Hsuan Lee, Gin Chang, Jun Ma, Guanyin Wang

**Affiliations:** ^1^Business School, Huaqiao University, Quanzhou, China; ^2^Department of Business Administration, National Central University, Taoyuan City, Taiwan; ^3^Management College, Ocean University of China, Qingdao, China

**Keywords:** broadcaster, leadership, cognitive loyalty, conative loyalty, self-construal, live streaming platform

## Abstract

Although considerable attention has been paid to the application of leadership in virtual communities, the field of live streaming has not been involved. This exploratory study aimed to explore how different broadcaster leadership traits (charismatic, authoritarian, and servant) influence audiences’ loyalty (cognitive and conative). And audience self-construal was chosen as a key moderator. The top 15 broadcasters from the regional rankings were selected from each of the two popular live streaming platforms, Douyu and YouTube, for the study. And we used snowball sampling with a link to an online questionnaire as a recruitment procedure. 310 audiences with live streaming experience from the Chinese Mainland and Taiwan participated. Hierarchical linear modeling was adopted for the analysis. This study found that broadcasters with servant and charismatic leadership traits positively affected cognitive loyalty. Broadcasters with servant leadership traits also had a positive effect on conative loyalty. Additionally, independent self-construal negatively moderated the relationship between servant leadership and cognitive loyalty. Independent self-construal positively moderated the relationship between authoritarian leadership and conative loyalty. Furthermore, interdependent self-construal negatively moderated the relationship between charismatic leadership and conative loyalty. Interdependent self-construal positively moderated the relationship between authoritarian leadership and conative loyalty. These conclusions extend the understanding of broadcasters’ traits and audiences’ psychology concerning the booming phenomenon of live streaming and can help platform managers motivate audiences’ loyalty on these platforms.

## Introduction

Such platforms, YouTube, as a new internet trend, live streaming, which involves high interactivity to meet the needs of consumers by presenting current real-time situations to end-users through Internet media, has gained great popularity (Fei et al., 2019). Live-stream broadcasters create content that results in a real-time interactive experience between the creator and the consumer and that differs from most forms of broadcast media (Sjoblom and Hamari, 2016). Since a live stream can also be interactive, it has some of the characteristics of a virtual community. Audiences and broadcasters can form virtual social relations in the process of interaction, so live streaming platforms are classified as social media platforms (Chen and Lin, 2018). The integration of live video into existing social media giants such as Facebook, Twitter, Instagram, and YouTube speak to the rising popularity of this content form (Savage, 2016). More than 10 million videos were broadcast during New Year’s Day in 2018, and over 100 million people watched the most-viewed live video (Facebook, 2018). Compared with the flourishing development of Facebook Live, the academic realm has paid unequal attention to live streaming activity. Limited studies have shed light on the formation and behavior of broadcasters (Hilvert-Bruce et al., 2018; Zhao et al., 2018; Fei et al., 2019; Zhou et al., 2019; Sun et al., 2020) but have not focused on the role of broadcasters. In the process of interaction, the role played by the leading party will influence the needs and satisfaction of the following party, loyalty, emotion, and the cohesion and formation of virtual community (Smith et al., 2013; Hamilton et al., 2014; Hu et al., 2017; Todd and Melancon, 2018; Yu et al., 2018). The role of the broadcaster in a live streaming is similar to that of the leader in an organization. Robbins (2003) demonstrated that leadership is the ability to influence a group to achieve a vision or goal. The source of this ability may be either formal, from the management class in the organization, or informal, from outside of an organization. Informal influence is as important as a formal influence, if not more so. During a live streaming, the behavior, expression and content of the broadcaster are examined by the audience. With the increase in the number of live streaming times, after a certain number of viewers and traffic are accumulated, supporters and opponents begin to appear. At this time, the audience is similar to the followers of a leader, and the broadcaster obtains informal leadership force. Although leadership on other social media, such as virtual communities and social networks, has received sufficient attention in the academic domain (Alhabash et al., 2015; Johnson and Woodcock, 2017; Weeks et al., 2017; Martínez and Olsson, 2018), leadership has not been considered in the field of live streaming. Therefore, this study applies leadership theory to broadcasters and classifies leadership style into three characteristics: charismatic leadership, authoritarian leadership, and servant leadership. Specifically, a broadcaster with charismatic leadership (BCL) tends to challenge different traditional modes of live streaming and enables the audience to unconsciously promote his ideas by conveying his own vision (Conger et al., 2000; Robbins, 2003) a broadcaster with authoritarian leadership (BAL) is imposing, shows a lofty posture, and requires the audience to be absolutely obedient to him (Whyte and Silin, 1977; Farh and Cheng, 2000) and a broadcaster with servant leadership (BSL) considers the audience’s needs as the primary goal in the live streaming and upholds an attitude of fairness, morality and integrity to establish a long-term trust relationship with the audience (Spears and Lawrence, 2002; Barbuto and Wheeler, 2006; Liden et al., 2008). Thus, incorporating the leadership characteristics of broadcasters in the current study may be helpful to understand the effect of broadcaster differentiation.

It is noteworthy that both audiences and broadcasters have important, active roles in live streaming platforms. With the increase in the number of broadcasters available, the audience’s adherence to specific broadcasters gradually declines. If a broadcaster cannot obtain the audience’s continuous loyalty, it will be difficult for the broadcaster to increase the retention rate of the audience and form its own fan group (Reichheld and Schefter, 2000; Reichheld et al., 2000; Shim et al., 2001; Gans, 2002). Therefore, we consider loyalty an important indicator to evaluate the effectiveness of the influence of broadcasters. Based on the model of loyalty (Oliver, 1997), “loyalty” in this paper is further divided into two distinct aspects: cognitive and conative loyalty. Cognitive loyalty means that the broadcaster has attracted the audience’s attention and won their recognition. Conative loyalty means that the audience will continue to pay attention to the broadcaster. These two types of loyalty also reflect broadcasters’ work performance. We therefore expect to gain a better understanding of how the traits of broadcaster leadership affect audiences’ cognitive and conative loyalty.

Additionally, broadcasters facilitate audience-broadcaster relationships by providing quasi-social experiences (Solomon, 1983) with their different leadership traits (charismatic, authoritarian, or servant), implying a hierarchical relationship structure. The social identity theory of leadership (Hogg, 2001) demonstrates that the effectiveness of a leader–member relationship is built on how members identify their relationships and how they form self-concepts within these relationships. Not everyone wishes to lead or to feel in control all of the time. Individuals with an interdependent self-construal who tend to follow instead of leading perform better when working with those they feel to be superior to themselves rather than with those on a similar level. However, people with independent self-construal need others to serve them (Earley, 1989; Bornstein, 1992; Singelis, 1994; Avolio et al., 1999). An authoritative broadcaster who puts himself in an authoritative position may attract more dependent viewers than independent ones. However, a service broadcaster may better serve an independent audience rather than an interdependent audience. Thus, it is necessary to consider such self-construals in the broadcaster-audience relationship literature.

This study aims to investigate and gain a better understanding of the traits of broadcaster leadership and consumers’ cognitive and conative loyalty with self-construal as a moderating factor. This study has three potential contributions. First, rather than considering broadcasters as an indiscriminate group (Hu et al., 2017; Todd and Melancon, 2018), this study is the first to attempt to distinguish broadcasters by their leadership characteristics. We expect to better understand the differentiated influence of broadcasters with different leadership traits on the audience. Second, users’ continuance intention has been widely studied in the examination of the social media effect (Hu et al., 2017). However, the loyalty of audiences in live streaming has been neglected. Customer loyalty has always been one of the most important strategies for enterprise operation. The core of many marketing activities lies in developing, maintaining, or improving customers’ loyalty to their products or services (Kotler, 1984). Highly loyal customers will continue to patronize a particular product or service (Pritchard and Howard, 1997). The attention and willingness to pay attention reflected in cognitive loyalty and intentional loyalty are important indicators to verify the effectiveness of live streaming. We take loyalty as an important index to evaluate the influence of broadcasters in the current study. Third, user gratification has been proven to be the main factor for an audience to follow a celebrity on social media (Stefanone and Jang, 2007; Lee et al., 2009). Broadcasters with authoritarian leadership, who put themselves in an authoritative position, clearly do not meet the needs of a dependent audience, and broadcasters with servant leadership, who tend to follow the audience, cannot meet the needs of an independent audience. Obviously, the existing research is insufficient to explain the behavior of the audience during a live streaming. Based on social identity theory, we introduce self-construal into the current study in an attempt to further understand the influence of the audience’s characteristics on the live streaming social relationship.

## Literature Review and Hypotheses Development

### Charismatic Leadership of Broadcaster (BCL)

Weber (1947) drew upon the Greek word charisma to develop the prototype of charismatic leadership and demonstrated that charismatic leadership involves the interaction and relationship between leaders and followers. Leaders identify a revolutionary, ideal target, and their followers have strong belief (Conger and Kanungo, 1987). Charm leadership theory (Robbins, 2003) unifies several characteristics: (i) articulate vision: charismatic leaders have vision and goals and can communicate them to their subordinates in a simple and understandable way so that their subordinates can clearly receive the message and understand its importance; (ii) personal risk: charismatic leaders are willing to pay a high price, take a high risk or even sacrifice themselves when necessary; (iii) environmental sensitivity: charismatic leaders have a keen observation of the transformation of the dreamland, and when faced with difficulties or limited development space, they can actually measure the required resources to cope with these changes; (iv) sensitivity to the needs of subordinates: charismatic leaders have the ability to perceive subordinates, grasp the changes and emotions in subordinates’ psychological needs, and provide feedback in an appropriate manner; (v) non-traditional behaviors: charismatic leaders need to have a vision, break away from tradition and norms, be highly sensitive to the environment, be willing to take a high level of personal risk, and exhibit extraordinary behavior. These factors make charismatic leaders different from non-charismatic leaders.

Previous studies of charismatic leadership have mainly focused on the leaders of enterprises or government organizations (Shamir et al., 1993; Conger et al., 2000; Lin and Huang, 2018) and have noted that leaders who possess the characteristics of charismatic leadership can also be reflected in brand anthropomorphism. Hackley et al. (2012) demonstrated that celebrities in creative industries can construct their own visions and images to be worshipped. As an emerging creative industry, live streaming is highly interactive and real-time, which allows the broadcaster to clearly present his vision and purpose so that the audience can clearly receive the message and understand its importance; the broadcaster can then construct his vision and image to be worshiped. Based on charm leadership theory (Robbins, 2003), we believe that broadcasters with charismatic leadership traits mainly have the following characteristics: (i) they clearly show their vision of and purpose for live streaming so that the audience can clearly receive the information and understand its importance, and the broadcasters then construct their own vision and image to be admired; (ii) they have their own distinctive characteristics and style to which they continue to adhere, even if they cannot cater to everyone’s preferences. (iii) They add their own interpretation of content on popular current affairs, terms or events, which shows that they are not only sensitive to the environment but also able to show what the audience wants to watch and know what attracts the audience. (iv) They have the foresight to break away from the traditional framework of live streaming and build a new style in their own professional live streaming to attract a larger audience.

### Authoritarian Leadership of Broadcaster (BAL)

Authoritarian leadership is based on the absolute obedience of followers to those in power, which means unconditional obedience and dependence (Farh and Cheng, 2000). In addition, there is a clear hierarchy between the top and bottom of the organization; the leader has the supreme power and cannot be challenged by others (Redding, 1990). To build their own prestige, leaders will not show their intentions so that they can maintain their control over employees and expand the social power distance between themselves and their subordinates (Westwood, 1997). Because Chinese people are influenced by the Confucian idea of respecting their teachers and the orderly family values of the young and the old, authoritarian leaders are more likely to be found in the Chinese context (Bor-Shiuan, 1995). Therefore, Bor-Shiuan (1995) developed the concept of paternalistic leadership by combining the six behavioral modes of Whyte and Silin (1977) and the viewpoints of Redding (1990). Westwood (1997) subsequently proposed nine elements of paternalistic leadership of Chinese family businesses: (i) didactic leadership; (ii) non-specific intention; (iii) reputation building; (iv) protection of dominance; (v) political manipulation; (vi) patronage and nepotism; (vii) conflict diffusion; (viii) aloofness and social distance; and (ix) dialog ideal.

Authoritarian broadcasters are usually highly professional in their field and content and often express their own opinions to convince the audience to accept their views, thus achieving didactic leadership. In addition, to establish an unattainable image or lofty status, these broadcasters define themselves as key opinion leaders who do not allow others to question their opinions or who even attack audience members with different opinions, thus arousing the audience’s emotional reaction. When there is strong emotion in comments, the results of viral spread will be added to the content (Kirby, 2004; Phelps et al., 2004; Eckler and Bolls, 2011; Hagerstrom et al., 2014). These strong emotions, whether positive or negative, have an obvious positive relationship with the amounts of reprints and discussions of the article or the video and have a positive impact on the attention the broadcaster receives (Alhabash et al., 2015).

### Servant Leadership of Broadcaster (BSL)

Servant leaders take the needs of their subordinates as their primary consideration. To enable subordinates to exert their full potential to achieve organizational goals and self-realization, service leaders try their best to support their subordinates’ accomplishments (Greenleaf, 1977). The motivation of service leaders is not their own interests but for their subordinates to work hard for the subordinates’ interests. They want to achieve not only the goals of the organization but, more importantly, the goals desired by their subordinates (Ehrhart, 2004). In contrast to traditional leadership methods, service-oriented leadership is more likely to build strong, long-term relationships with integrity and sincerity (Graham, 1991). Liden et al. (2008) defined leaders with service leadership as having the following characteristics: (i) emotional healing; (ii) creating value for the community; (iii) conceptual skills; (iv) empowering; (v) helping subordinates grow and succeed; (vi) putting subordinates first; (viii) relationships; and (ix) servanthood.

Audiences tend to seek information directly related to their own interests and then consider the newsreader’s identity (Lin, 1992). Broadcasters with the qualities of servant leadership are similar to newsreaders; they need to face audiences and meet the audience’s needs. To obtain more affection from the audience, the broadcaster serves the public request and presents the content the audience wants to watch in the live streaming. These broadcasters serve the audience as their primary goal and even sacrifice themselves to make the audience happy. When communicating with other broadcasters, they can integrate the characteristics of their services into the team and empower junior broadcasters to add value to the team and establish a stable and long-term organizational relationship. In terms of personality traits, these broadcasters treat all people sincerely and keep their promises to the audience and other broadcasters.

### Loyalty and Broadcaster Leadership

Scholars have discussed many aspects of customer loyalty. One of the most important indicators of customer loyalty is whether customers have repeated purchases of a product or service. Through this indicator, customers can be divided into four groups: undivided, divided, unstable, no loyalty (Brown, 1952). In addition to this, the customer for a product or service attitude is also a big target, that is to say, the effective method to measure customer loyalty to both two aspects to behavior and attitude (Gremler, 1995). As for the measurement of attitude and behavior, the most widely accepted one is the interaction and trust between human beings (Gundlach and Murphy, 1993). This also indicates that there is a significant correlation between the customer’s trust in the brand and the brand loyalty (Lau and Lee, 1999). There is strong evidence to support this argument: the more customers trust a brand, the more loyal they will be to the brand and the more likely they will keep buying (Chaudhuri and Holbrook, 2001).

Dick and Basu (1994) demonstrated that customer loyalty is mainly reflected in customers’ attitude and repeated purchase behavior, which includes cognition, emotion and intention. On this basis, Oliver (1997) found that the establishment of loyalty begins from an internal attitude and then is demonstrated in behavior. Loyalty can be divided into four dimensions (Oliver, 1999): (i) cognitive loyalty, which refers to the loyalty generated by customers through rational thought after receiving relevant information on products or services; (ii) affective loyalty, which refers to customers’ attitude toward a product or service based on their own consultation and relevant experience; (iii) conative loyalty, which refers to customers’ willingness to purchase a product or service again; and (4) action loyalty, which refers to the fact that customers will overcome the obstacles that may hinder their purchase and convert their intention into actual purchase behavior.

Previous research has shown that when audiences watch TV, they will want to interact with other audiences (Lee and Andrejevic, 2014), which is called social TV engagement. The phenomenon of social TV engagement adds an interactive layer for simple program watching, provides audience social space, and shares group knowledge and information with a wide range of audiences (Gross et al., 2008; Lee and Andrejevic, 2014). Audiences will also be more impressed with the program or channel because of the interaction, thus improving its popularity (Nagy and Midha, 2014; Lim et al., 2015; Lin et al., 2018).

In addition, when customers participate in virtual interaction related to brand advertising, they can create brand loyalty and purchase intention through social presence (Kang et al., 2013) and commitment (Sahoo et al., 2010). Their perception of brand characteristics is a key driving factor for building customer loyalty (Gustafsson et al., 2005; Sashi, 2012; Kang et al., 2013). Today, broadcasters can interact with audiences in real-time to create a social presence and emotional commitment, which is helpful for broadcasters to establish their own brand loyalty.

With the development of science and technology, users no longer need to interact with each other on two screens. Mobile terminals allow broadcasters to contact audiences through any channel at any time and place during live streaming and to respond to audiences in real-time. The benefits of social TV engagement can be applied to consolidate or enhance audience loyalty. In addition, brand loyalty is highly correlated with brand characteristics. The broadcasters of live streaming are similar to brands. In the face of fierce competition, it is necessary to have unique brand characteristics and distinct leadership characteristics. However, regardless of the leadership traits a broadcaster has, his ultimate goal is to attract more viewers and increase their stickiness and loyalty (Sjoblom and Hamari, 2016). Therefore, in this paper, we explore the relationship between broadcaster leadership and loyalty.

In combination with the characteristics of live streaming and the definition of loyalty (Oliver, 1997), in this paper, we use cognitive loyalty and conative loyalty to discuss the behavior of the live audience. Audiences show cognitive loyalty when they are attracted to and recognize broadcasts. When the audience has the intention to follow the broadcaster and to continue to subscribe to the broadcaster, they show their conative loyalty.

Audiences, as passive recipients of information, are vulnerable to the subjective opinions of broadcasters, who can influence antagonistic relations between different groups (Anastasio et al., 1999). Changes in broadcasters’ behaviors also lead to changes in audiences’ behaviors. Therefore, we further explore the influence of broadcasters with different leadership traits on cognitive loyalty and conative loyalty.

Charismatic leaders are highly contagious and can quickly influence the audience’s preferences and psychological state. In addition, charismatic leaders can quickly integrate into the group, shorten the distance and become popular with the public (Ryu et al., 2007; Cocker and Cronin, 2017). Generally, these broadcasters show enthusiasm and present positive and optimistic images in live streaming. Due to their sensitivity to information and the environment, they can attract audiences with relatively new information, so it is easier for them to generate cognitive loyalty. Broadcasters with the characteristics of authoritarian leadership like to demonstrate their authority in front of the audience, and their method of live streaming is very imposing (Cheng et al., 2000; Shu et al., 2018). They are believed to be more able to control the overall situation and live broadcasts (Ertureten et al., 2013). Therefore, it is easier for the audience to fully accept the broadcaster’s preference, which can leave a deep impression on the audience. In terms of the audience’s contact with the live streaming, they will think that the broadcaster performs well on the whole, thus generating cognitive loyalty.

The servant leadership qualities of a broadcaster have a strong moral sense. The moral foundation of these broadcasters is based on service motivation (Sousa and Van Dierendonck, 2017). They are committed to promoting participation in ethical behavior that benefits others or groups. While spreading high moral standards, they also attract normative people (González and Guillén, 2008).

Through these acts, service leaders can instill a sense of moral integrity and obligation into the organization, increasing normative commitment (González and Guillén, 2008; Meyer and Parfyonova, 2010; Lapointe and Vandenberghe, 2015). Therefore, for a broadcaster with servant leadership qualities, initial contact with the audience is important for a good impression. The audience will feel that the broadcast content is good, which produces cognitive loyalty toward broadcasters.

Thus, we suggest the following hypotheses:

H1: Different leadership traits of broadcaster have significant impact on audiences’ cognitive loyalty.
  H1a: *BCL has a significant impact on audiences’ cognitive loyalty.*
  H1b: *BAL has a significant impact on audiences’ cognitive loyalty.*
  H1c: *BSL has a significant impact on audiences’ cognitive loyalty.*

Conative loyalty refers to the willingness of customers to purchase a product or service again (Oliver, 1997). Internet celebrities, such as pop stars, are considered excellent representatives. Broadcasters give new meaning to celebrities. Compared with traditional celebrities, broadcasters can bring a sense of reality to the audience through more real-time interaction so that the audience will like them and subscribe to gain more opportunities to interact with the broadcasters (Jerslev, 2016; Shin, 2016). The number of subscribers is an important indicator to measure the performance of a broadcaster. When viewers like a broadcaster, they will subscribe to the live stream as a way of real-time tracking to continuously follow the live streaming of the broadcaster. Sjoblom and Hamari (2016) suggest that audiences subscribe to strengthen their connection with broadcasters and other audiences. Lee and Kim (2017) note that subscription indicates increased audience affection for the broadcaster, which can increase the intention to donate. Therefore, this study suggests that when viewers subscribe, they express conative loyalty toward broadcasters.

Broadcasters with charismatic leadership traits can connect followers and give meaning to joint efforts and goals (Marks et al., 2001; Varella et al., 2012). They promote group identity and reciprocal cooperation (Shamir and Howell, 1999; Varella et al., 2012), and followers therefore show more obedience (Deluga, 1995; Den Hartog et al., 2007). This inspires followers to participate in the vision and promote organizational development and organizational citizenship behavior (Deluga, 1995). Civic behavior in an organization is a kind of spontaneous behavior, such as automatically encouraging the broadcaster through donation, indicating that the donor is a strong fan of the broadcaster and will always support him among all broadcasters. This process shows the audience’s conative loyalty toward a broadcaster. However, broadcasters with authoritarian leadership traits will constantly emphasize personal dominance (Tsui et al., 2004). When interacting with the audience, these broadcasters make important decisions or commands on the spot. The audience will be divided according to the degree of completion. These broadcasters will deliberately raise the standard of good audiences to continuously improve their status and maintain distance from the audience (Schuh et al., 2013). In the process of information transmission, broadcasters with authoritarian characteristics tend to transmit information in one direction during live streaming and do not accept other voices in an attempt to build their own prestige. When recipients passively receive the message, they accept the distorted view. In the process of acceptance, they establish social identity in their own social group (Anastasio et al., 1999). The opinions of the audience are influenced by the other members of the group, which is a powerful force, far more powerful than any force outside the group (Mackie et al., 1990). At this time, audiences who like the broadcaster will express their conative loyalty by subscribing to express that they are good audiences in the broadcaster’s mind and to demonstrate their sense of identity to the group. Broadcasters with the characteristics of service-oriented leadership give subordinates more autonomy and decision-making power and provide resources to help them develop their skills and abilities (Van Dierendonck and Nuijten, 2011; Chiniara and Bentein, 2016; Chughtai, 2019). Such positive actions may improve employees’ self-efficacy and satisfy subordinates’ demand for autonomy (Chiniara and Bentein, 2016). Self-determination theory (Andersen et al., 2000) shows that meeting basic human needs for autonomy can increase employees’ intrinsic motivation (Chughtai, 2019). A broadcaster with the characteristics of servant leadership can increase the intrinsic motivation of the audience by giving them the right to decide what to broadcast, thus strengthening the audience’s motivation to continue to follow the broadcaster. Audiences are willing to voluntarily continue to subscribe, demonstrating conative loyalty. Therefore, we suggest the following hypotheses:

H2: Different leadership traits of broadcaster have significant impact on audiences’ conative loyalty.
  H2a: *BCL has a significant impact on audiences’ conative loyalty.*
  H2b: *BAL has a significant impact on audiences’ conative loyalty.*
  H2c: *BSL has a significant impact on audiences’ conative loyalty.*

### The Moderate Effect of Self-Construal

Prior research has demonstrated that followers’ personal characteristics, emotions and attitudes influence their perceptions of or preferences for certain traits of relationships with superiors and their propensity to follow a particular trait of a leader (Ehrhart and Klein, 2001; Kark et al., 2003). Kim et al. (2010) confirmed that individual differences have a significant impact on the stickiness of social media. The social identity theory of leadership (Hogg, 2001) suggests that the effectiveness of a leader–member relationship is built on how members identify their relationships and how they form self-concepts within these relationships.

The social cognitive theory of mass communication (Bandura, 2001) has developed a sustainable theoretical construct to demonstrate self-construal and self-related behaviors. A self-construal can be defined as a collection of feelings, actions and thoughts concerning the self; it is the way that people think about and define themselves and how they relate to the wider world (Triandis, 1989; Singelis, 1994). In this article, we investigate two aspects of self-construal, interdependent and independent. An interdependent self-construal (ITD) is conceptualized as an individual’s perception of themselves as having a variable, flexible self. Individuals with an interdependent self-construal believe they are intertwined with others and are impressionable; they can be molded in situations. In contrast, an independent self-construal (ID) is conceptualized as the feeling that an individual has a “unitary stable, bounded” self. Individuals with an independent self-construal are relatively separate from their social context; they often express themselves directly and directly say what they think. They are unlikely to be heavily influenced by others’ feelings or actions and do not readily change their thinking (Markus and Kitayama, 1991; Singelis, 1994).

Independent people tend to be self-interested when working in a team. They often welcome anyone who can contribute to their need for personal development and are motivated by useful people (Avolio et al., 1999; Burroughs and Rindfleisch, 2002). Therefore, when broadcasters with charismatic leadership traits show outstanding professional skills, reputation and charm to be admired in a live streaming (Conger and Kanungo, 1987), independent people will feel that these broadcasters will bring benefits to them, and they will develop cognitive loyalty toward the charismatic broadcasters. In contrast, broadcasters with service characteristics respect the thoughts and opinions of fans (Liden et al., 2008). These broadcasters can meet the needs of independent audiences who like to express themselves directly (Singelis, 1994) and generate cognitive loyalty toward charismatic broadcasters. Authoritative broadcasters like audiences who do what the broadcaster tells them to do, and they are used to putting themselves in positions of absolute authority (Cheng et al., 2000). Independent people instinctively tend to place a high priority on personal goals when working in a team. They generally dislike being told what to do (Avolio et al., 1999; Burroughs and Rindfleisch, 2002). Therefore, it is difficult for such broadcasters to attract independent audiences and generate cognitive loyalty. Additionally, independent viewers are unlikely to change their minds as they spend more time with broadcasters and have more interaction experience (Markus and Kitayama, 1991; Singelis, 1994). They usually maintain a unitary, stable, bounded self. Therefore, we suggest the following hypotheses:

H3: ID moderates the relationship between different leadership traits of broadcaster and audiences’ cognitive loyalty.
  H3a: *ID moderates the relationship between BCL and audiences’ cognitive loyalty.*
  H3b: *ID moderates the relationship between BAL and audiences’ cognitive loyalty.*
  H3c: *ID moderates the relationship between BSL and audiences’ cognitive loyalty.*

H4: ID moderates the relationship between different leadership traits of broadcaster and audiences’ conative loyalty.
  H4a: *ID moderates the relationship between BCL and audiences’ conative loyalty.*
  H4b: *ID moderates the relationship between BAL and audiences’ conative loyalty.*
  H4c: *ID moderates the relationship between BSL and audiences’ conative loyalty.*

Interdependent people are expected to readily identify with their superiors’ goals or the common goals of the group. Bornstein (1992) demonstrated that interdependent people tend to follow others with alacrity and seek out dependent relationships with superiors. They perform well when working with superiors and present high levels of loyalty (Earley, 1989; Avolio et al., 1999). Broadcasters with charismatic leadership traits can express a clear vision and are willing to lead fans (Conger and Kanungo, 1987) and make them part of the vision. This process can satisfy the needs of the interdependent audience for belonging and integration (Singelis, 1994) and make them like the broadcaster. Therefore, this study suggests that interdependent traits can enhance the audience’s cognitive loyalty toward charismatic broadcasters. In contrast, broadcasters with authoritarian leadership display self-confidence, which has an impact on their followers. They tend to provide a vision that can reduce uncertainty and fear by clearly defining situations (Hollander, 1992). Respect for and increased conformity to leaders increases interdependent followers’ confidence in their choices (Shamir et al., 1993). Therefore, when watching the live streaming of authoritarian broadcasters, an interdependent audience will like them and express cognitive loyalty. Broadcasters with the characteristics of service leadership are good at showing empathy by listening to and understanding the feelings and needs of others (Spears and Lawrence, 2002). They adopt the psychological perspective of their followers and show warmth, compassion, and forgiveness in their relationships, a trait that helps create a brotherly, compassionate, and trusting atmosphere (Van Dierendonck and Nuijten, 2011; Linuesa-Langreo et al., 2017). This enables a dependent audience to obtain a sense of belonging (Singelis, 1994) and thus to hold a positive attitude toward the service broadcaster. Therefore, we expect that interdependent traits can strengthen an audience’s cognitive loyalty to a service broadcaster.

An interdependent self-construal (ITD) is conceptualized as an individual’s perception of themselves as having a variable, flexible self. With the increase in the cognition of broadcasters and the increase in interaction time, the feelings of an interdependent audience toward the characteristics of broadcasters and the resulting loyalty relationship may change. For example, in the early stage, a dependent audience may develop cognitive loyalty because of considerate service broadcasters. However, because this kind of audience tends to follow instead of leading, the audience performs better when working with those they feel to be superior to themselves rather than those on a similar level (Earley, 1989; Singelis, 1994; Avolio et al., 1999). Therefore, with the accumulation of interactive experience, the interdependent audience may express greater loyalty intention toward authoritative broadcasters than toward service broadcasters. In summary, we make the following inferences:

H5: ITD moderates the relationship between different leadership traits of broadcaster and audiences’ cognitive loyalty.
  H5a: *ITD moderates the relationship between BCL and audiences’ cognitive loyalty.*
  H5b: *ITD moderates the relationship between BAL and audiences’ cognitive loyalty.*
  H5c: *ITD moderates the relationship between BSL and audiences’ cognitive loyalty.*

H6: ITD moderates the relationship between different leadership traits of broadcaster and audiences’ conative loyalty.
  H6a: *ITD moderates the relationship between BCL and audiences’ conative loyalty.*
  H6b: *ITD moderates the relationship between BAL and audiences’ conative loyalty.*
  H6c: *ITD moderates the relationship between BSL and audiences’ conative loyalty.*

**FIGURE 1:**
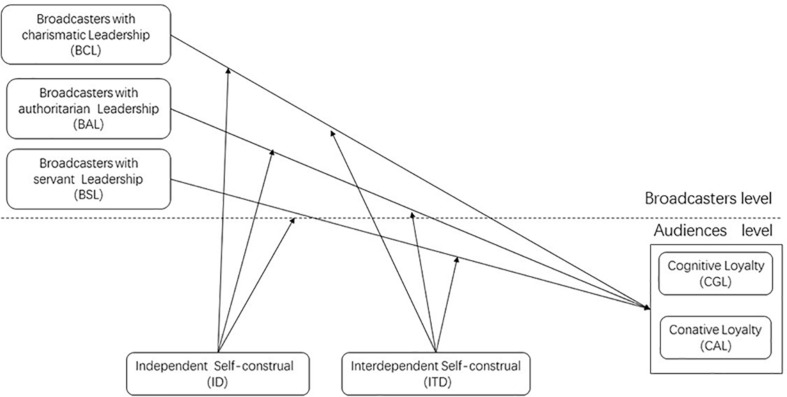
The conceptual model.

Figure 1 illustrates the conceptual model guiding our research hypotheses.

## Methodology

### Participants and Procedures

A web-based survey was used to collect data for quantitative testing of the study model in Chinese Mainland and Taiwan. Specifically, the selection of popular platforms refers to Love Game (2017). Two live streaming platforms, Douyu and YouTube, were selected as the study objects. Audiences of platforms were recruited according to their actual experiences (they must have at least 6 months of experience watching live streaming) and were required to finish the website link of a questionnaire. Based on this, we used snowball sampling with a link to an online questionnaire as recruitment procedure. Snowball sampling is based on referrals made among audiences. Each platform lists the top 15 popular broadcasters and generates a list of optional broadcasters in the questionnaires. If the subject chooses the option “other,” it means there is no favorite broadcaster in the list, which should be regarded as an invalid questionnaire. After selecting favorite broadcaster, participants were asked to answer the remaining questions in the questionnaire based on their previous experience watching that broadcaster. In order to avoid repeated filling by the same participant, the single IP address can be filled only once. As an incentive, participants were offered a fast-food restaurant coupon.

The survey was conducted from April 20 to Jun 30, 2020. Of the 310 electronic questionnaires, 262 were completed and returned. Forty-eight questionnaires were incomplete and not used, leaving the number of valid questionnaires at 262 (Response rate = 84.51%). There is no significant difference in usage time and willingness to participate between two platforms after *T*-test analysis. We also compared the first quarter with the last quarter of the questionnaire and the difference is still not significant. The male-to-female ratio was approximately 55.73 to 44.27. Most of the respondents were aged 18–34 years old (85.1%), young people account for a larger proportion of the total population. A majority of respondents (60.9%) were students, followed by service employees (15.1%). More than 73.1% of respondents have been watching live streaming for more than half a year and more than 70.4% of respondents watched the broadcast more than twice a week. We pretest the valence of questioner of 66 respondents. The questionnaire was revised based on the results of the pretest.

### Measurement

The questionnaire includes five parts. Participants were asked to choose their favorite broadcasters in Part 1. Appendix A show the list of broadcasters we provided to participants to choose from. Part 2 is based on the constructs of BCL, BAL, and BSL. We adapted Conger and Kanungo (1987) 11-item scale (α = 0.86) to measure BCL, Cheng et al. (2000) 13-item scale (α = 0.90) to measure BAL and Liden et al.’s (2008) 7-item scale (α = 0.76) to measure BSL. Part 3 is based on the construct of loyalty. To measure cognitive loyalty and conative loyalty, the 8-item scale developed by Harris and Goode (2004) was used (cognitive loyalty scale: α = 0.91; conative loyalty scale: α = 0.88). Part 2 and Part 3 are 7-point Likert scales with 1 representing strongly disagree and 7 representing strongly agree. Part 4 is based on the construct of self-construal. We adapted 24 items from Singelis (1994). The scale consisted of two dimensions: Interdependent self-construal (α = 0.83) and Independent self-construal (α = 0.77). 5-point Likert scales with 1 representing strongly disagree and 5 representing strongly agree in this part. The aim of Part 5 is to measure control variables, Prior research studies have indicated that customers’ gender, age, education, occupation, length of watching, average week usage time, average daily usage time, and use device influence audience perception and behavior (Guo et al., 2016; Hu et al., 2017). Thus, the aforementioned variables were listed as control variables for testing the hypotheses of audience level. Items from Part 2 to Part 5 are presented in Table 1.

**TABLE 1:** Questionnaire items.

Variable	Measurement	Source
**Part 2**		
**Broadcaster leadership traits**		
Broadcaster with charismatic leadership (BCL)	(1) This broadcaster is essentially opposed to status quo and strives to change the mode, content and so on.	Conger and Kanungo (1987)
	(2) The live mode of this broadcaster is not limited to the traditional mode.	
	(3) This broadcaster is expert in using unconventional means to transcend the existing live content or mode.	
	(4) This broadcaster has strong articulation of future vision and is willing to lead fans.	
	(5) This broadcaster pursues idealized vision which is highly discrepant from status quo (number of viewers, popularity, concept, etc.).	
	(6) The shared perspective and idealized vision by this broadcaster make fans consider him/her as a likable and honorable hero worthy of identification and imitation.	
	(7) This broadcaster is willing to selflessly take his/her own risks to defend the rights and interests of fans.	
	(8) In order to change the status quo, this broadcaster is very sensitive to the external trend or fans.	
	(9) This broadcaster has certain professional skills, reputation and admirable charm.	
	(10) The image of this broadcaster is very outstanding, and he/she is the fan’s model.	
	(11) This broadcaster can influence fans and make them unconsciously promote his/her idea or popularity.	
Broadcaster with authoritarian leadership (BAL)	(1) Audiences are required to fully follow the opinions of this broadcaster.	Cheng et al., 2000
	(2) When audiences or other broadcasters object to the opinions of this broadcaster in public, they will be satirized by him/her.	
	(3) Good audiences in the eyes of this broadcaster must obey him/her.	
	(4) Events during the live broadcast are almost at the discretion of this broadcaster.	
	(5) When the live streaming is interrupted (such as the interruption of live streaming due to equipment	
	problems, etc.), the final decision will be made based on the opinions of this broadcaster.	
	(6) This broadcaster does not disclose any information irrelevant to the live streaming to audiences.	
	(7) It is not easy for the audience to perceive the real intention of this broadcaster.	
	(8) When face to audiences, this broadcaster always shows the professional authority.	
	(9) The live streaming mode of this broadcaster is very powerful.	
	(10) This broadcaster adopts a serious way to live.	
	(11) When the audience utters malicious words, this broadcaster will scold the audience.	
	(12) This broadcaster stressed that the audience in the chat room must abide by the order.	
	(13) When other broadcasters violate the rules, this broadcaster will express dissatisfaction.	
Broadcaster with servant leadership (BSL)	(1) I would seek help from this broadcaster if I had a personal problem.	Liden et al., 2008
	(2) This broadcaster emphasizes the importance of giving back to fans.	
	(3) When fans make mistakes, swear or disturb the order, this broadcaster will correct them in time.	
	(4) This broadcaster will respect fans’ ideas and opinions.	
	(5) This broadcaster makes fans’ needs a priority.	
	(6) This broadcaster puts fans’ best interests ahead of his/her own.	
	(7) This broadcaster would not compromise ethical principles in order to get fans’ subscription and reward.	
**Part 3**		
**Loyalty**		
Cognitive loyalty	(1) As far as my contact with live streaming is concerned, I think he/she is still good as a whole.	Harris and Goode, 2004
	(2) As far as my contact with live streaming is concerned, I think the content (the theme of discussion and sharing, the content of performance, etc.) of live streaming of this broadcaster is good.	
	(3) As far as my contact with live streaming is concerned, the content (the theme of discussion and sharing, the content of performance, etc.) of live streaming of this broadcaster is quite in line with my hobbies, interests, and tastes.	
	(4) As far as my contact with live streaming is concerned, the way (reply to information, reply to audience requests, etc.) this broadcaster interacts with the audience is good.	
Conative loyalty	(1) On the whole, I often feel that this broadcaster is the best.	
	(2) I seldom feel that this broadcaster has a bad performance or a bad situation.	
	(3) I don’t think the live streaming performance of this broadcaster is as good as it was at the beginning, and it is going from bad to worse.	
	(4) In my heart, I often feel that the live content (the theme of discussion and sharing, the content of performance, etc.) of this broadcaster is the best.	
**Part 4**		
**Self-construal**		
Interdependent self-construal (ITD)	(1) I have respect for the authority figures with whom I interact.	Singelis, 1994
	(2) It is important for me to maintain harmony within me group.	
	(3) My happiness depends on the happiness of those around me.	
	(4) I would offer my seat in a bus to elders.	
	(5) I respect people who are modest about themselves.	
	(6) I will sacrifice my self-interest for the benefit of the group I am in.	
	(7) I often have the feeling that my relationships with others are more important than my own.	
	(8) I should take into consideration my parents’ advice when making education/career plans.	
	(9) It is important to me to respect decisions made by the group.	
	(10) I will stay in a group if they need me, even when I’m not happy with the group.	
	(11) If my brother or sister fails, I feel responsible.	
	(12) Even when I strongly disagree with group members, I avoid an argument.	
Independent self-construal (ID)	(1) I’d rather say “No” directly, than risk being misunderstood.	
	(2) Speaking up during a class is not a problem for me.	
	(3) Having a lively imagination is important to me.	
	(4) I am comfortable with being singled out for praise or rewards.	
	(5) I am the same person at home that I am at school.	
	(6) Being able to take care of myself is a primary concern for me.	
	(7) I act the same way no matter who I am with.	
	(8) I feel comfortable using someone’s first name soon after I meet them, even when they are with people I’ve just met.	
	(9) I prefer to be direct and forthright when dealing with people I’ve just met 22. I enjoy being unique and different from others in many respects.	
	(10) My personal identity independent of others, is very important to me.	
	(11) I value being in good health above everything.	
**Part 5**		
**Control variables**		
(1) Gender: male, female.		Guo et al., 2016; Hu et al., 2017
(2) Age: under 13 years old, 13–17 years old, 18–24 years old, 25–34 years old, 35–44 years old, 45–54 years old, 55 years old or above.		
(3) Education: junior high school or below, senior high school/vocational high school, university/college, master degree or above.		
(4) Occupation: housewife/househusband, white-collar workers, students, senior white-collar workers/boss, retired/unemployed, freelancer.		
(5) Length of watching: less than 6 months, 6–12 months, 12–24 months, 3–4 years, 5–6 years, more than 6 years		

### Analysis Strategy

In this study, because participants provided data at the team level (level-2 – leadership trait of broadcaster) and the individual level (level-1-loyalty and self-construal), our hypotheses were examined by cross-level techniques. Since HLM can deal with non-independence issues and estimate the influences of the different level factors on the dependent variables (Raudenbush and Bryk, 2002), HLM was applied to examine the hypotheses in this study. This study adopted grand mean-centered techniques for all level-1 (individual level) variables in accordance with the suggestion of Hofmann and Gavin (1998) to examine all the hypotheses. Furthermore, we employed the product of coefficients tests to examine whether mediation effects existed (MacKinnon et al., 2002). We further used the PRODCLIN program to estimate the confidence interval of the indirect effect (MacKinnon et al., 2007).

## Results

### Psychometric Characteristics of the Measures

Table 2 summarizes the descriptive statistics of the variables used in this study. And seven confirmatory factor analyses (CFA) were computed using AMOS 20.0 to test the measurement models. The model-fit measures were used to assess the model’s overall goodness of fit (GFI, NFI, AGFI, and RMR) and values all exceeded their respective common acceptance levels (Hair et al., 2006).

**TABLE 2:** Descriptive statistics of the variables.

Variables	Mean	Standard deviation	Reliability
**Team level**			
BCL	3.90	0.50	0.84
BAL	2.70	0.69	0.89
BSL	3.73	0.52	0.83
**Individual level**			
CGL	4.13	0.58	0.76
CAL	3.64	0.60	0.81
ITD	3.70	0.44	0.83
ID	3.73	0.46	0.80

The convergent validity of scale items was calculated on the basis of reliability, composite reliability, and average variance extracted (AVE) (Fornell and Larcker, 1981). Only the CGL and CAL of AVE exceeded the benchmark of 0.50 recommended by Hair et al. (2006). However, Hair et al. (2006, p. 808) also suggested a more loosely standard that the value of AVE exceeded 0.25 is acceptable. Similarly, Bagozzi and Yi (1988) pointed out that it is difficult to meet the standard (i.e., AVE > 0.50), and suggested that the three-fourths of AVE values exceeded 0.50 is acceptable due to difficulty meeting the standard in practice. According to Fornell and Larcker (1981), when the average variance extracted estimates are below 0.5 and composite reliability are above 0.6, the scale still has convergent validity. In addition, Bentler and Wu (1993) also pointed out that AVE should be over 0.20 in each latent variable. Thus, the scales used for the present study have convergent validity.

For GFI, in addition to BCL, BAL, ITD, ID below the standard value of 0.9, other variables were in line with the standard. However, according to the evaluation standard of Hu and Bentler (1999), GFI > 0.8 is an acceptable range.

In terms of NFI, BSL, CGL, and CAL were above the standard value of 0.9. But according to the evaluation standard of Hu and Bentler (1999), NFI > 0.8 is an acceptable range.

In terms of AGFI, although the values for all variables were range from 0.8 to 0.9, according to the research of Baumgartner and Homburg (1996), the proportion of AGFI lower than the standard value of 0.8 is 48%, but still has a good adaptability.

In terms of RMR, BAL, ITD and ID were all above the standard value of 0.05. However, according to the evaluation standard of Hu and Bentler (1999), RMR below 0.08 is an acceptable range. In addition, according to the evaluation standard of Floyd and Widaman (1995), RMR below 0.1 is also considered as an acceptable range. In general, the result showed that the measurement model exhibited a close fit with the collected data (see Table 3).

**TABLE 3:** Convergent validity.

Variables	AVE	CR	GFI	NFI	AGFI	RMR
BCL	0.324	0.834	0.869	0.862	0.803	0.047
BAL	0.371	0.867	0.870	0.833	0.806	0.072
BSL	0.388	0.774	0.971	0.942	0.923	0.030
CGL	0.679	0.966	0.930	0.933	0.894	0.024
CAL	0.638	0.954	0.929	0.912	0.893	0.032
ITD	0.300	0.833	0.886	0.807	0.825	0.061
ID	0.259	0.803	0.890	0.765	0.825	0.058

We estimated discriminant validity by comparing the common variance between factors with the AVE among the six factors (Fornell and Larcker, 1981). Table 4 shows that the constructs met this criterion. Hence, discriminant validity was assured. In sum, the six constructs met the standards of reliability, convergent validity, and discriminant validity.

**TABLE 4:** Discriminant validity.

Variables	CGL	CAL	ITD	ID
CGL	0.679^*a*^			
CAL	0.308**	0.638^*a*^		
ITD	0.138**	0.166**	0.278^*a*^	
ID	0.125**	0.062**	0.027**	0.217^*a*^

^*a*^Represents the square root of AVE, and the other matrix entries are the correlation coefficient. **p ≤ 0.01.

To examine the appropriateness of the data aggregation of the level 1 (audience level), we computed *r*_wg_ values of leadership traits of broadcaster (level 2 – broadcaster level) to examine the inter-rater agreement. The results showed that the large values for charismatic, authoritarian, and servant leadership were 0.95, 0.89, and 0.93. The *r*_wg_ values were higher than 0.7 (the accepted level) (James et al., 1984); therefore, aggregating the responses of leadership trait of broadcaster at the unit-level proceeded.

### Tests of Hypotheses

At first, in order to assess the appropriateness of the multilevel analysis, the current study estimated a null model, which means no predictors in the audience level (level 1) and the broadcaster level (level 2), to estimate the significance level of the group level. The results revealed significant between-group variance (χ^2^[32] = 44.759, *p* = 0.001) for cognitive loyalty. Also, the results revealed significant between-group variance (χ^2^[32] = 40.715, *p* = 0.001) for conative loyalty. Therefore, a multilevel analysis was appropriate for the data.

Table 5 shows the results of Hypotheses 1–2. The results revealed that BCL was positively related to CGL (γ = 0.537, *p* = 0.001; see M1). And BSL was positively related to CGL (γ = 0.447, *p* = 0.001; see M3). However, CAL had not significant effect on CGL (γ = −0.027, *p* = 0.782; see M2). Thus, the result supported H1. Specifically, the result supported H1a and H1c, but not H1b. Additionally, the result revealed that only BSL was positively related to CAL (γ = 0.571, *p* = 0.001; see M12). BCL had not significant effect on CAL (γ = 0.302, *p* = 0.117; see M10). Meanwhile, BAL had not significant effect on CAL (γ = 0.007, *p* = 0.947; see M11). Thus, the result supported H2. Specifically, the result supported H2c, but not H2a, H2b.

**TABLE 5:** The HLM results of broadcaster leadership and loyalty.

	CGL				CAL			
Dependent variable	Null model	M1	M2	M3	Null model	M10	M11	M12
**Audience level**								
Intercept	4.125	2.550	4.682	2.820	3.642	2.875	4.003	1.720
	(0.043)	(0.663)	(0.406)	(0.720)	(0.034)	(0.805)	(0.411)	(0.625)
	***	***	***	***	***	***	***	**
Gender		0.168	0.208	0.221		−0.006	0.018	0.036
		(0.074)	(0.074)	(0.066)		(0.066)	(0.068)	(0.056)
		*	**	**				
Age		−0.144	−0.145	−0.134		−0.003	0.003	0.017
		(0.029)	(0.030)	(0.028)		(0.036)	(0.037)	(0.037)
		***	***	***				
Education Level		−0.070	−0.073	−0.058		−0.108	−0.108	−0.089
		(0.037)	(0.038)	(0.038)		(0.066)	(0.067)	(0.061)
Length of watching		0.014	0.004	0.007		−0.015	−0.027	−0.020
		(0.021)	(0.021)	(0.022)		(0.035)	(0.033)	(0.031)
**Broadcaster level**								
BCL		0.537				0.302		
		(0.151)				(0.156)		
		***						
BAL			−0.027				0.006	
			(0.098)				(0.090)	
BSL				0.447				0.571
				(0.163)				(0.135)
				**				***
Within-team variance σ^2^	0.325	0.288	0.294	0.291	0.356	0.332	0.330	0.318
Between-team variance τ_00_	0.013	0.180	0.027	0.017	0.000	0.526	0.533	0.321
Deviance	458.681	439.062	449.056	439.696	475.291	483.226	487.016	473.202

*p ≤ 0.05, **p ≤ 0.01, ***p ≤ 0.001.

**FIGURE 2:**
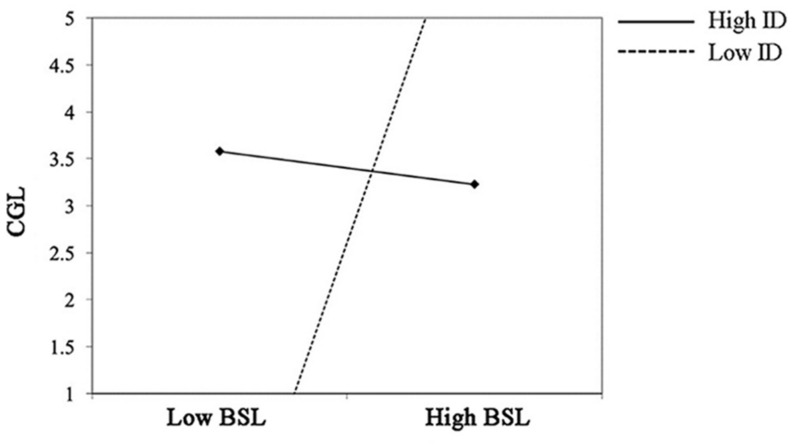
Interaction between BSL and ID on CGL.

Table 6 shows the results of Hypotheses 3–4. The result demonstrated that ID negatively moderated the relationship between BSL and CGL (γ = −0.404, *p* = 0.004; see M8). In other words, when facing the broadcasters with a high quality of servant leadership, the audience with a low quality of independent self-construal will have a high level of cognitive loyalty (see Figure 2).

**TABLE 6:** The HLM results of broadcaster leadership, ID and loyalty.

	CGL				CAL			
Dependent variable	Null	M4	M6	M8	Null	M13	M15	M17
**Audience level**								
Intercept	4.125	4.554	3.585	−4.319	3.642	2.953	5.686	2.094
	(0.043)	(3.990)	(1.283)	(1.897)	(0.034)	(4.860)	(1.400)	(3.539)
	***		**	*	***		***	
Gender		0.181	0.213	0.225		0.012	0.039	0.044
		(0.072)	(0.073)	(0.067)		(0.059)	(0.061)	(0.052)
		**	**	**				
Age		−0.137	−0.134	−0.125		0.014	0.025	0.032
		(0.028)	(0.029)	(0.026)		(0.038)	(0.040)	(0.037)
		***	***	***				
Education Level		−0.089	−0.086	−0.072		−0.126	−0.110	−0.098
		(0.047)	(0.047)	(0.048)		(0.070)	(0.070)	(0.062)
Length of watching		0.003	−0.003	−0.005		−0.035	−0.038	−0.035
		(0.022)	(0.022)	(0.022)		(0.036)	(0.032)	(0.030)
**Broadcaster level**								
ID		−0.440	0.308	2.029		0.118	−0.495	−0.056
		(1.002)	(0.320)	(0.504)		(1.368)	(0.391)	(0.944)
				***				
BCL		−0.423				−0.007		
		(1.014)				(1.222)		
BAL			−0.300				−1.066	
			(0.468)				(0.473)	
							*	
BSL				1.854				0.186
				(0.507)				(0.909)
				***				
BCL*ID		0.236				0.049		
		(0.259)				(0.350)		
BAL*ID			0.070				0.296	
			(0.114)				(0.129)	
							*	
BSL*ID				−0.404				0.091
				(0.130)				(0.243)
				**				
Within-team variance σ^2^	0.325	0.244	0.247	0.243	0.356	0.296	0.292	0.290
Between-team variance τ_00_	0.013	0.614	0.299	0.311	0.000	1.858	1.622	1.012
Deviance	458.681	403.484	412.141	400.982	475.291	468.383	470.310	460.277

*p ≤ 0.05, **p ≤ 0.01, ***p ≤ 0.001.

But independent self-construal did not moderate the relationship between BCL and CGL (γ = 0.236, *p* = 0.369; see M4). Also, independent self-construal did not moderate the relationship between BAL and CGL (γ = 0.070, *p* = 0.542; see M6). Thus, the result supported H3. Specifically, the result supported H3c, but not H3a and H3b.

**FIGURE 3:**
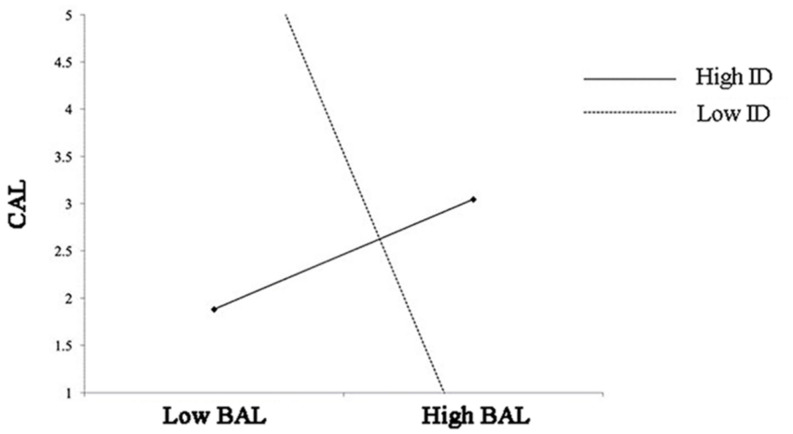
Interaction between BAL and ID on CAL.

Besides, the result revealed that ID positively moderated the relationship between BAL and CAL (γ = 0.296, *p* = 0.028; see M15). In other words, when confronted with the broadcasters with high authoritarian leadership, the audience with high independent self-construal would have higher conative loyalty (see Figure 3).

But ID did not moderate the relationship between BCL and CAL (γ = 0.049, *p* = 0.890; see M13). Also, ID did not moderate the relationship between BSL and CGL (γ = 0.091, *p* = 0.708; see M15). Thus, the result supported H4. Specifically, the result supported H4b, but not H4a and H4c.

Table 7 shows the results of Hypotheses 5–6. The result demonstrated that ITD did not moderate the relationship between BCL and CGL (γ = 0.037, *p* = 0.864; see M5). Also, we investigated the same effect on BAL (γ = 0.134, *p* = 0.248; see M7) and BSL (γ = 0.062, *p* = 0.781; see M9). Thus, the result did not support H5 (H5a, H5b, and H5c).

**TABLE 7:** The HLM results of broadcaster leadership, ITD and loyalty.

	CGL				CAL			
Dependent variable	Null	M5	M7	M9	Null	M14	M16	M18
**Audience level**								
Intercept	4.125	1.339	4.293	2.181	3.642	8.569	5.559	0.912
	(0.043)	(3.177)	(1.349)	(3.262)	(0.034)	(3.740)	(1.182)	(2.939)
	***		**		***	*	***	
Gender		0.125	0.172	0.188		0.030	−0.012	0.008
		(0.060)	(0.059)	(0.055)		(0.052)	(0.054)	(0.047)
		*	**	**				
Age		−0.125	−0.120	−0.116		0.019	0.023	0.025
		(0.028)	(0.029)	(0.028)		(0.033)	(0.035)	(0.035)
		***	***	***				
Education level		−0.060	−0.060	−0.051		−0.082	−0.090	−0.078
		(0.041)	(0.042)	(0.042)		(0.063)	(0.064)	(0.060)
Length of watching		0.025	0.017	0.017		0.000	−0.018	−0.010
		(0.019)	(0.018)	(0.018)		(0.030)	(0.029)	(0.028)
**Broadcaster level**								
ITD		0.305	0.085	0.207		3.141	−0.446	0.263
		(0.828)	(0.351)	(0.834)		(1.046)	(0.353)	(0.756)
						**		
BCL		0.400				2.832		
		(0.818)				(0.974)		
						**		
BAL			−0.546				−1.105	
			(0.456)				(0.419)	
							**	
BSL				0.162				0.414
				(0.863)				(1.245)
BCL*ITD		0.037				−0.709		
		(0.214)				(0.271)		
						*		
BAL*ITD			0.134				0.297	
			(0.114)				(0.118)	
							*	
BSL*ITD				0.062				0.026
				(0.220)				(0.200)
Within-team variance σ^2^	0.325	0.241	0.244	0.244	0.356	0.288	0.287	0.283
Between-team variance τ_00_	0.013	0.614	0.284	0.420	0.000	0.108	0.093	0.057
Deviance	458.681	402.884	413.693	405.940	475.291	456.999	462.807	452.181

*p ≤ 0.05, **p ≤ 0.01, ***p ≤ 0.001.

**FIGURE 4:**
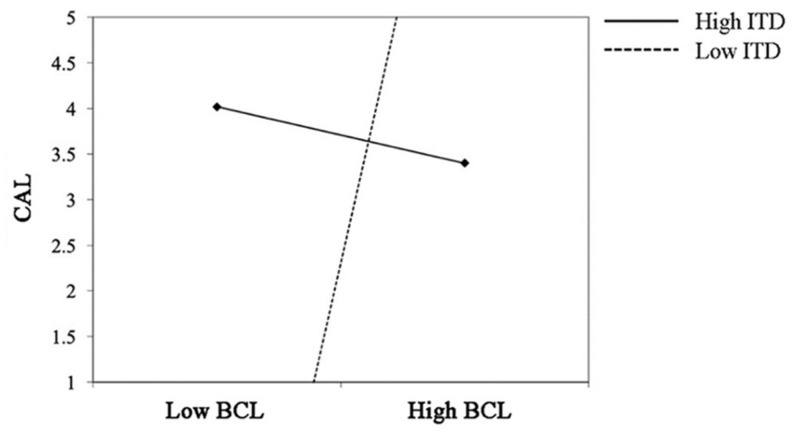
Interaction between BCL and ITD on CAL.

Furthermore, ITD negatively moderated the relationship between BCL and CAL (γ = −0.709, *p* = 0.014; see M14). In other words, when facing the broadcasters with high charismatic leadership, the audience with low independent self-construal will have high conative loyalty (see Figure 4).

**FIGURE 5:**
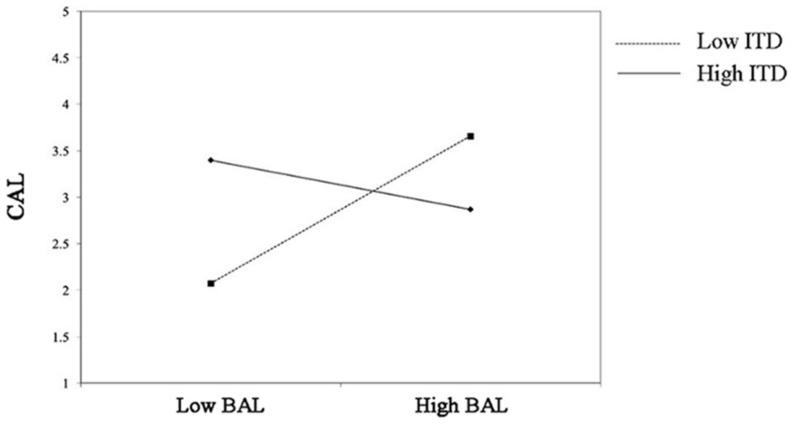
Interaction between BAL and ITD on CAL.

And ITD positively moderated the relationship between BAL and CAL (γ = 0.297, *p* = 0.017; see M16). When faced with a broadcaster with high authoritarian leadership, the audience with high interdependent self-construal would have a higher level of conative loyalty (see Figure 5).

**FIGURE 6:**
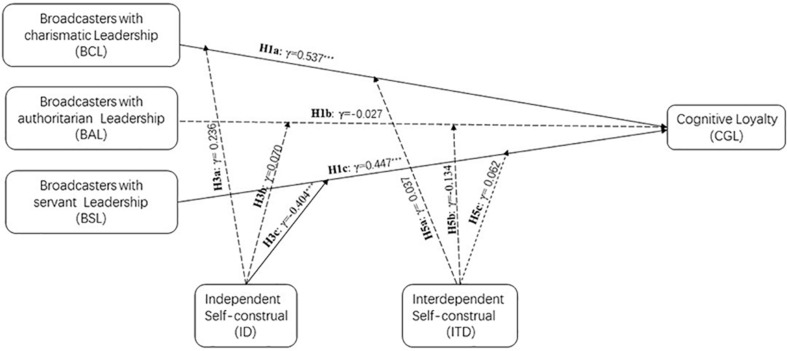
Effect of broadcaster’s leadership and cognitive loyalty with self-construal as moderate.

**FIGURE 7:**
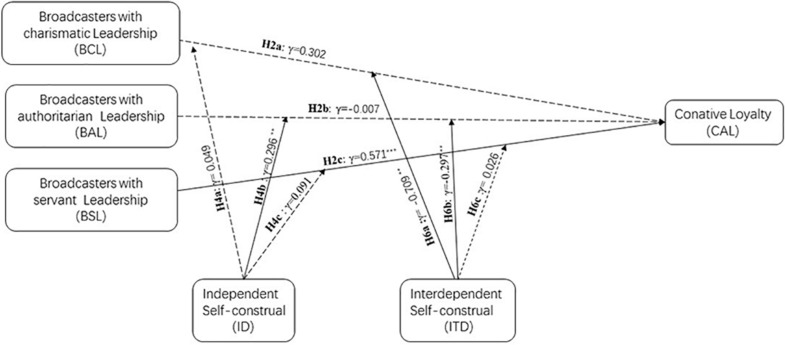
Effect of broadcaster’s leadership and conative loyalty with self-construal as moderate.

But ITD moderated the relationship between BSL and CAL (γ = 0.026, *p* = 0.898; see M18). Thus, the result supported H6. Specifically, the result supported H6a, H6b, but not H6 (see Figures 6, 7).

## General Discussion

This study developed an integrated model to investigate the factors that influence audience loyalty in live streaming platforms. The results indicate that the traits of broadcasters’ leadership affect audiences’ cognitive and conative loyalty. In particular, broadcasters with the characteristics of service-oriented leadership have a positive and significant influence on both cognitive loyalty and action loyalty. The results further confirm that service leaders are humble, willing to listen and share, and value the quality of service (Carter and Baghurst, 2013; Van Dierendonck and Patterson, 2014). Charismatic broadcasters also have a positive and significant relationship with cognitive loyalty. From these findings, we can see that charismatic broadcasters are highly contagious and can attract the attention of the audience (Conger and Kanungo, 1987; Conger et al., 2000).

We did not observe a main effect of broadcasters with authoritarian leadership on responses to cognitive loyalty and conative loyalty. This rules out other potential confounds associated with political manipulation and social distance. In an organization, an authoritarian leader will clearly distinguish the hierarchy between the top and bottom. The leader has supreme power and cannot be challenged by others, while employees should fully follow the instructions of the authoritarian leader and accept his authority; employees thus lose their own autonomy (Redding, 1990). To further establish their own prestige and to be Machiavellian, leaders will not indicate their intentions to maintain their control over employees and expand the social power distance between themselves and their subordinates (Westwood, 1997). Authoritarian broadcasters will make the audience feel manipulated by the information and instructions they present unilaterally. Moreover, to maintain their own prestige, they will deliberately keep a certain distance from the audience, which contrasts with the original intention of the audience to pursue social connection on the social platform (Westwood, 1997). Therefore, although authoritarian leaders may leave a deep impression on the audience, they may not be recognized by the audience, and the audience may not be willing to pay attention continuously. Therefore, these leaders cannot have an impact on cognitive loyalty and conative loyalty.

Additionally, differences in broadcaster leadership with regard to audiences cause self-construal to play a systematic role in determining consumer responses to cognitive loyalty and conative loyalty. The independent self-construal of an audience has a negative influence on the audience’s cognitive loyalty when they are faced with a service broadcaster, but it has a positive influence on conative loyalty when the audience faces an authoritative broadcaster. When audiences face authoritarian broadcasters, independent self-construal has a negative influence on conative loyalty.

### Theoretical Implications

The current study contributes to the extant literature on live streaming research in the following ways. First, previous discussion of the celebrity effect on social media platforms was mainly limited to (i) film clips and contents of specific emotions or themes; (ii) specific broadcasters and network celebrities; and (iii) the gender, appearance, content and behavior of broadcasters and network celebrities, which influence the audience (Lin, 1992; Anastasio et al., 1999; Alhabash et al., 2015; Johnson and Woodcock, 2017; Martínez and Olsson, 2018). Leadership on other social media, such as virtual communities and social networks, has received sufficient attention in academic domains (Alhabash et al., 2015; Johnson and Woodcock, 2017; Weeks et al., 2017; Martínez and Olsson, 2018), but it has not been considered in the field of live streaming. Thus, the style of the broadcaster and the role of the broadcaster in the interactive relationship have not been explored in previous studies. Our study confirmed that broadcasters’ different leadership styles and roles have different effects on audiences’ cognitive loyalty and conative loyalty. Thus, it can be demonstrated that leadership theory can be used to explain the behavior of a broadcaster during live streaming and its relationship with audience loyalty.

Second, the core of enterprise marketing activities often lies in the development, maintenance, or improvement of customers’ loyalty to products or services (Kotler, 1984). Loyal customers will continue to patronize a particular product or service, so managers attach great importance to customer loyalty (Pritchard and Howard, 1997). With the rapid development of online consumption, many scholars believe that it is more difficult to sell online than offline, and it is more difficult to build online loyalty than offline loyalty (Reichheld and Schefter, 2000; Reichheld et al., 2000). As an increasingly important online activity, live streaming is often discussed with regard to the audience’s willingness to continue watching (Hu et al., 2017; Zhao et al., 2018; Fei et al., 2019), but as an important indicator of long-term relationship building, loyalty has been neglected. The results of this study indicate that through different levels of loyalty, the psychological changes and behaviors of the audience can be observed. Different traits of broadcasters have different influences on audiences’ levels of loyalty.

Third, this study contributes important insights to the literature on self-construal. Although existing research has attempted to propose different reasons why social media activities may affect consumers’ choice at the theoretical level (Wilcox and Stephen, 2013; Weiger et al., 2018), little research has examined these mechanisms empirically. Based on the social cognitive theory of mass communication (Bandura, 2001), this study contributes to the discourse on the important role of self-construal in the relation between broadcaster leadership and audience loyalty. These findings help to better understand how social media activities affect consumer thinking styles; thus, the findings help us to understand the influence of audience characteristics on live streaming social relationships.

### Practical Implications

Based on the findings of this study, we recommend that broadcasters should adjust their content and method of live streaming according to their own leadership characteristics to gain more loyal audiences. For authoritarian broadcasters, they should introduce the characteristics of service-oriented leadership as much as possible and know how to listen to the audience’s feelings and needs. The tone of live streaming can still be tough, but the audience should feel the broadcasters’ kindness. Charismatic broadcasters should attach importance to establishing a long-term trust relationship with the audience to promote the audience’s behaviors of active subscription and donation. Although the enthusiasm of charismatic broadcasters is very appealing, the content of the live streaming often abandons the old and establishes the new, demonstrating a new style. It is difficult for an audience to internalize new ideas in a short time and generate identification. Therefore, in the process of delivering consultation, these broadcasters should pay attention to good guidance and to the needs of most people.

Moreover, live streaming fosters authenticity, visualization, and interactivity in online shopping. Taking note of the power of live streaming, international brands such as Guess, Clarks, and Anna Sui (e.g., fashion shows) have used e-commerce live to reach their customers. The way broadcasters present products and brands become the key to determining sales performance in live streaming. If the brand style is consistent with the spokesperson style, the credibility of advertising information transmission can be increased, and then the brand attitude of consumers can be affected (Pradhan et al., 2016). When broadcasters promote brands to consumers on live broadcast platforms, they are playing the role of brand spokesmen. Therefore, if the brand style matches the leadership style of the broadcaster (e.g., Apple matches the broadcaster with authoritative leadership), we can use the results to understand the mechanism of variation in audience’s loyalty.

Also, operator of live streaming platforms should change their marketing strategy. Most operators usually recommend broadcasters to audiences based on the subscription number, without considering the individual characteristics of the audiences. Instead, operators can predict the personality characteristics of customers by collecting their behavioral data (such as the number of interactions and duration of stay) in the live broadcast, so as to recommend more accurate broadcasters. If the audience seldom speaks after entering the chat room but they pay attention for a long time, they can be classified as an audience with a low degree of independence. On this basis, more service broadcasters can be recommended. However, if the audience is eager to express their opinions after entering the studio and they often have the intention to attract others’ attention (such as rewarding the broadcaster before and after the speech to attract the attention of the broadcaster), they can be classified as an audience with low interdependence traits. On this basis, more charismatic broadcasters can be recommended.

The personality of the audience is also worthy of the broadcaster’s attention. In combination with the leadership characteristics of the broadcasters themselves, corresponding responses are given to audiences with different characteristics. Usually, audiences with independent self-construal are not afraid to speak out actively and present themselves generously. What they hope to gain is the recognition and affirmation of the broadcasters rather than material satisfaction. They will also attach great importance to whether broadcasters have their own opinions. If broadcasters blindly follow a trend, it will be difficult for them to gain the attention and respect of this type of audience. We suggest that broadcasters should first listen more to understand what the audience is trying to express and then combine this with the speech they are going to make to give the audience timely recognition. After accumulating certain feelings and credibility, audience members will naturally have the opportunity to actively subscribe and donate. Audiences with interdependent self-construal will attach excessive importance to the atmosphere of the chat room and the harmony of the whole process of living conditions. They also value the connection among audience members. When broadcasters show the characteristics of being responsible and reliable, this will help to build a sense of belonging to the group and meet the needs of a dependent audience to promote the behaviors of active subscription and donation.

### Limitations and Future Research

First, this study did not consider the role of another important group in live streaming, namely, the influence of other audiences who interact with each other. Specifically, the entirety of live streaming content is not just provided by broadcasters. Contributions from multiple audiences complement the overall experience of watching live streaming content and distinguish it from the consumption of traditional TV programs (Smith et al., 2013). Thus, we could further explore the influence of co-experience with other audiences in the relationship of broadcasters’ leadership and loyalty. Second, regarding the proposed framework and selected constructs, future studies might explore additional moderators to develop a more holistic understanding of live streaming consumption. Audiences’ identification with broadcasters has been proven to be positively associated with their continuous watching intention (Hu et al., 2017). The influence of audience identification with broadcasters on loyalty should be included to further augment the proposed framework. Third, different types are presented by live streaming platforms in different countries. For example, TV series are the most preferred content type streamed, followed by sports, tutorials, gaming, social in the United States. Although the content of Chinese live streaming platforms covers a wide range of types, they mainly focus on shows, games, and social. Compared with social type, tutorial type of living streaming is more likely to have a leader-subordinate relationship between the broadcaster and audiences. Thus, we could further explore the influence of content type in the relationship of broadcasters’ leadership and loyalty. Fourth, it is acknowledged that the study data collection process might restrict the generalizability of the results. Our survey mainly covers areas dominated by Asian culture. Future studies are recommended to extend the current study scope to include other cultural areas. Finally, although demographic variables of the audience were not the main factor we explored in this study, we did find that audience age and gender had a significant impact on cognitive loyalty. Therefore, in future studies, we will further observe the demographic variables of the audience.

## Data Availability Statement

The raw data supporting the conclusions of this article will be made available by the authors, without undue reservation.

## Ethics Statement

The studies involving human participants were reviewed and approved by Ethics Committee of the National Central University. The patients/participants provided their written informed consent to participate in this study.

## Author Contributions

YH and YL contributed to the improvement of the initial idea. YH and GW designed the study and collected the data. GC and YH conducted the analysis. GC, YH, and JM worked on the first draft. JM and YH revised the conceptualizations and the final manuscript. All authors contributed to the article and approved the submitted version.

## Conflict of Interest

The authors declare that the research was conducted in the absence of any commercial or financial relationships that could be construed as a potential conflict of interest.
