# Corrigendum: Broadcasters' Leadership Traits and Audiences' Loyalty With the Moderating Role of Self-Construal: An Exploratory Study

**DOI:** 10.3389/fpsyg.2021.744920

**Published:** 2021-08-30

**Authors:** Yidan Huang, Yi Hsuan Lee, Gin Chang, Jun Ma, Guanyin Wang

**Affiliations:** ^1^Business School, Huaqiao University, Quanzhou, China; ^2^Department of Business Administration, National Central University, Taoyuan City, Taiwan; ^3^Management College, Ocean University of China, Qingdao, China

**Keywords:** broadcaster, leadership, cognitive loyalty, conative loyalty, self-construal, live streaming platform

In the original article, there was an error in affiliation 2 as published. Instead of “School of Management, National Central University, Taoyuan City, Taiwan”, it should be “Department of Business Administration, National Central University, Taoyuan City, Taiwan”.

The authors apologize for this error and state that this does not change the scientific conclusions of the article in any way. The original article has been updated.

## Publisher's Note

All claims expressed in this article are solely those of the authors and do not necessarily represent those of their affiliated organizations, or those of the publisher, the editors and the reviewers. Any product that may be evaluated in this article, or claim that may be made by its manufacturer, is not guaranteed or endorsed by the publisher.

