# Supplementary figures and images for: Broadcasters’ Leadership Traits and Audiences’ Loyalty With the Moderating Role of Self-Construal: An Exploratory Study
Source: Front Psychol. 2021 Apr 22;12:605784. doi: 10.3389/fpsyg.2021.605784 (PMC8100459; doi:10.3389/fpsyg.2021.605784)

**APPENDIX A**

**
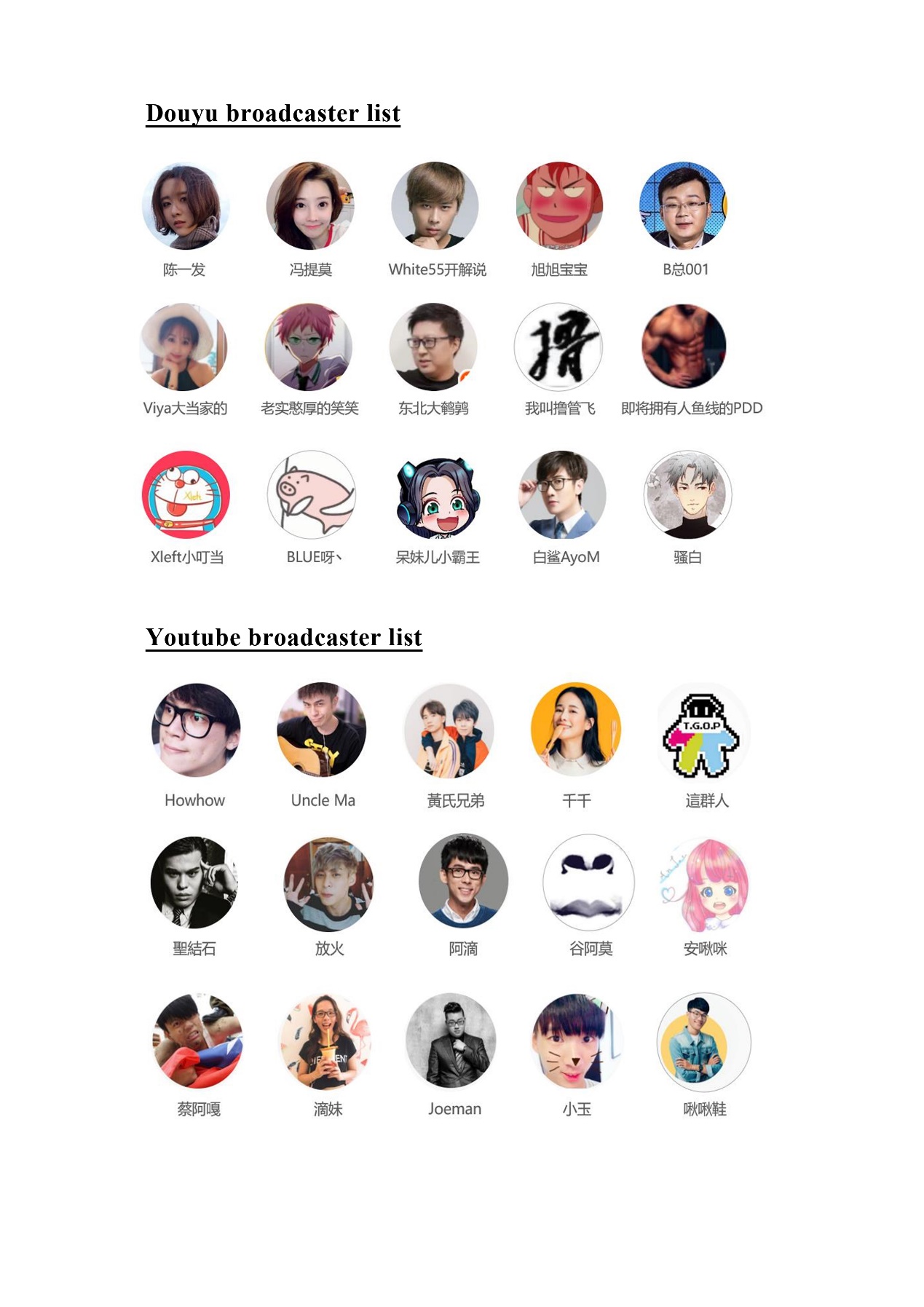
**

Supplement: Supplementary file 1 [file Data_Sheet_1.docx]
