# Supplementary material for: Broadcasters’ Leadership Traits and Audiences’ Loyalty With the Moderating Role of Self-Construal: An Exploratory Study
Source: Front Psychol. 2021 Apr 22;12:605784. doi: 10.3389/fpsyg.2021.605784 (PMC8100459; doi:10.3389/fpsyg.2021.605784)
Supplement: Supplementary file 2 [file Table_1.docx]

**Table 1.** Questionnaire items

| **Variable** | **Measurement** | **Source** |
| --- | --- | --- |
| **Part 2** | | |
| ***Broadcaster Leadership Traits*** | | |
| Broadcaster with  Charismatic Leadership  (BCL) | 1. This broadcaster is essentially opposed to status quo and strives to change the mode, content and so on. 2. The live mode of this broadcaster is not limited to the traditional mode. 3. This broadcaster is expert in using unconventional means to transcend the existing live content or mode. 4. This broadcaster has strong articulation of future vision and is willing to lead fans. 5. This broadcaster pursues idealized vision which is highly discrepant from status quo (number of viewers, popularity, concept, etc.). 6. The shared perspective and idealized vision by this broadcaster make fans consider him/her as a likable and honorable hero worthy of identification and imitation. 7. This broadcaster is willing to selflessly take his/her own risks to defend the rights and interests of fans. 8. In order to change the status quo, this broadcaster is very sensitive to the external trend or fans. 9. This broadcaster has certain professional skills, reputation and admirable charm. 10. The image of this broadcaster is very outstanding, and he/she is the fan's model. 11. This broadcaster can influence fans and make them unconsciously promote his/her idea or popularity. | Conger and Kanungo (1987) |
| Broadcaster with  Authoritarian Leadership  (BAL) | 1. Audiences are required to fully follow the opinions of this broadcaster. 2. When audiences or other broadcasters object to the opinions of this broadcaster in public, they will be satirized by him/her. 3. Good audiences in the eyes of this broadcaster must obey him/her. 4. Events during the live broadcast are almost at the discretion of this broadcaster. 5. When the live streaming is interrupted (such as the interruption of live streaming due to equipment problems, etc.), the final decision will be made based on the opinions of this broadcaster. 6. This broadcaster does not disclose any information irrelevant to the live streaming to audiences. 7. It is not easy for the audience to perceive the real intention of this broadcaster. 8. When face to audiences, this broadcaster always shows the professional authority. 9. The live streaming mode of this broadcaster is very powerful. 10. This broadcaster adopts a serious way to live. 11. When the audience utters malicious words, this broadcaster will scold the audience. 12. This broadcaster stressed that the audience in the chat room must abide by the order. 13. When other broadcasters violate the rules, this broadcaster will express dissatisfaction. | Bor-Shiuan, Li-fang and Jing-Lih (2000) |
| Broadcaster with  Servant Leadership (BSL) | 1. I would seek help from this broadcaster if I had a personal problem. 2. This broadcaster emphasizes the importance of giving back to fans. 3. When fans make mistakes, swear or disturb the order, this broadcaster will correct them in time. 4. This broadcaster will respect fans' ideas and opinions. 5. This broadcaster makes fans’ needs a priority. 6. This broadcaster puts fans’ best interests ahead of his/her own. 7. This broadcaster would not compromise ethical principles in order to get fans' subscription and reward. | Liden et al. (2008) |
| **Part 3** | | |
| ***Loyalty*** | | |
| Cognitive loyalty | 1. As far as my contact with live streaming is concerned, I think he/she is still good as a whole. 2. As far as my contact with live streaming is concerned, I think the content (the theme of discussion and sharing, the content of performance, etc.) of live streaming of this broadcaster is good. 3. As far as my contact with live streaming is concerned, the content (the theme of discussion and sharing, the content of performance, etc.) of live streaming of this broadcaster is quite in line with my hobbies, interests and tastes. 4. As far as my contact with live streaming is concerned, the way (reply to information, reply to audience requests, etc. ) this broadcaster interacts with the audience is good. | Harris and Goode (2004) |
| Conative loyalty | 1. On the whole, I often feel that this broadcaster is the best. 2. I seldom feel that this broadcaster has a bad performance or a bad situation. 3. I don't think the live streaming performance of this broadcaster is as good as it was at the beginning, and it is going from bad to worse 4. In my heart, I often feel that the live content (the theme of discussion and sharing, the content of performance, etc.) of this broadcaster is the best. |  |
| **Part 4** | | |
| ***Self-Construal*** | | |
| Interdependent Self-Construal  (ITD) | 1. I have respect for the authority figures with whom I interact. 2. It is important for me to maintain harmony within me group. 3. My happiness depends on the happiness of those around me. 4. I would offer my seat in a bus to elders. 5. I respect people who are modest about themselves. 6. I will sacrifice my self-interest for the benefit of the group I am in. 7. I often have the feeling that my relationships with others are more important than my own. 8. I should take into consideration my parents' advice when making education/career plans. 9. It is important to me to respect decisions made by the group. 10. I will stay in a group if they need me, even when I'm not happy with the group. 11. If my brother or sister fails, I feel responsible. 12. Even when I strongly disagree with group members, I avoid an argument. | Singelis (1994) |
| Independent Self-Construal  (ID) | 1. I'd rather say "No" directly, than risk being misunderstood. 2. Speaking up during a class is not a problem for me. 3. Having a lively imagination is important to me. 4. I am comfortable with being singled out for praise or rewards. 5. I am the same person at home that I am at school. 6. Being able to take care of myself is a primary concern for me. 7. I act the same way no matter who I am with. 8. I feel comfortable using someone's first name soon after I meet them, even when they are with people I've just met. 9. I prefer to be direct and forthright when dealing with people I've just met22. I enjoy being unique and different from others in many respects. 10. My personal identity independent of others, is very important to me. 11. I value being in good health above everything. |  |
| **Part 5** | | |
| ***Control Variables*** | | |
| 1. Gender: Male, Female 2. Age: Under 13 years old, 13-17 years old, 18-24 years old, 25-34 years old, 35-44 years old, 45-54 years old, 55 years old or above 3. Education: Junior high school or below, Senior high school / Vocational high school, University / College, Master degree or above 4. Occupation: Housewife/Househusband, White-collar workers, Students, Senior white-collar workers/Boss, Retired/Unemployed, Freelancer 5. Length of watching: Less than 6 months, 6-12 months, 12-24 months, 3-4 years, 5-6 years, More than 6 years | | Guo et al. (2016); Hu et al.( 2017) |

**Table 2.** Descriptive statistics of the variables.

| Variables | Mean | Standard deviation | Reliability |
| --- | --- | --- | --- |
| *Team level* | | | |
| BCL | 3.90 | 0.50 | 0.84 |
| BAL | 2.70 | 0.69 | 0.89 |
| BSL | 3.73 | 0.52 | 0.83 |
| *Individual level* | | | |
| CGL | 4.13 | 0.58 | 0.76 |
| CAL | 3.64 | 0.60 | 0.81 |
| ITD | 3.70 | 0.44 | 0.83 |
| ID | 3.73 | 0.46 | 0.80 |

**Table 3.** Convergent validity.

| Variables | AVE | CR | GFI | NFI | AGFI | RMR |
| --- | --- | --- | --- | --- | --- | --- |
| BCL | 0.324 | 0.834 | 0.869 | 0.862 | 0.803 | 0.047 |
| BAL | 0.371 | 0.867 | 0.870 | 0.833 | 0.806 | 0.072 |
| BSL | 0.388 | 0.774 | 0.971 | 0.942 | 0.923 | 0.030 |
| CGL | 0.679 | 0.966 | 0.930 | 0.933 | 0.894 | 0.024 |
| CAL | 0.638 | 0.954 | 0.929 | 0.912 | 0.893 | 0.032 |
| ITD | 0.300 | 0.833 | 0.886 | 0.807 | 0.825 | 0.061 |
| ID | 0.259 | 0.803 | 0.890 | 0.765 | 0.825 | 0.058 |

**Table 4.** Discriminant validity.

| Variables | CGL | CAL | ITD | ID |
| --- | --- | --- | --- | --- |
| CGL | 0.679^a^ |  |  |  |
| CAL | 0.308^**^ | 0.638 ^a^ |  |  |
| ITD | 0.138^**^ | 0.166^**^ | 0.278^a^ |  |
| ID | 0.125^**^ | 0.062^**^ | 0.027^**^ | 0.217^a^ |

^a^ Represents the square root of AVE, and the other matrix entries are the correlation coefficient.

**p≤.01.

**Table 5.** The HLM results of broadcaster leadership and loyalty.

|  | CGL | | | | CAL | | | |
| --- | --- | --- | --- | --- | --- | --- | --- | --- |
| Dependent variable | Null model | M1 | M2 | M3 | Null model | M10 | M11 | M12 |
| *Audience level* | | | | |  |  |  |  |
| Intercept | 4.125 | 2.550 | 4.682 | 2.820 | 3.642 | 2.875 | 4.003 | 1.720 |
|  | (0.043) | (0.663) | (0.406) | (0.720) | (0.034) | (0.805) | (0.411) | (0.625) |
|  | *** | *** | *** | *** | *** | *** | *** | ** |
| Gender |  | 0.168 | 0.208 | 0.221 |  | -0.006 | 0.018 | 0.036 |
|  |  | (0.074) | (0.074) | (0.066) |  | (0.066) | (0.068) | (0.056) |
|  |  | * | ** | ** |  |  |  |  |
| Age |  | -0.144 | -0.145 | -0.134 |  | -0.003 | 0.003 | 0.017 |
|  |  | (0.029) | (0.030) | (0.028) |  | (0.036) | (0.037) | (0.037) |
|  |  | *** | *** | *** |  |  |  |  |
| Education Level |  | -0.070 | -0.073 | -0.058 |  | -0.108 | -0.108 | -0.089 |
|  |  | (0.037) | (0.038) | (0.038) |  | (0.066) | (0.067) | (0.061) |
| Length of watching |  | 0.014 | 0.004 | 0.007 |  | -0.015 | -0.027 | -0.020 |
|  |  | (0.021) | (0.021) | (0.022) |  | (0.035) | (0.033) | (0.031) |
| *Broadcaster level* | | | | |  |  |  |  |
| BCL |  | 0.537 |  |  |  | 0.302 |  |  |
|  |  | (0.151) |  |  |  | (0.156) |  |  |
|  |  | *** |  |  |  |  |  |  |
| BAL |  |  | -0.027 |  |  |  | 0.006 |  |
|  |  |  | (0.098) |  |  |  | (0.090) |  |
| BSL |  |  |  | 0.447 |  |  |  | 0.571 |
|  |  |  |  | (0.163) |  |  |  | (0.135) |
|  |  |  |  | ** |  |  |  | *** |
| Within-team variance σ^2^ | 0.325 | 0.288 | 0.294 | 0.291 | 0.356 | 0.332 | 0.330 | 0.318 |
| Between-team variance τ_00_ | 0.013 | 0.180 | 0.027 | 0.017 | 0.000 | 0.526 | 0.533 | 0.321 |
| Deviance | 458.681 | 439.062 | 449.056 | 439.696 | 475.291 | 483.226 | 487.016 | 473.202 |

*p≤.05.

**p≤.01.

***p≤.001.

**Table 6.** The HLM results of broadcaster leadership, ID and loyalty.

|  | CGL | | | | CAL | | | |
| --- | --- | --- | --- | --- | --- | --- | --- | --- |
| Dependent variable | Null | M4 | M6 | M8 | Null | M13 | M15 | M17 |
| *Audience level* | | | | | | | | |
| Intercept | 4.125 | 4.554 | 3.585 | -4.319 | 3.642 | 2.953 | 5.686 | 2.094 |
|  | (0.043) | (3.990) | (1.283) | (1.897) | (0.034) | (4.860) | (1.400) | (3.539) |
|  | *** |  | ** | * | *** |  | *** |  |
| Gender |  | 0.181 | 0.213 | 0.225 |  | 0.012 | 0.039 | 0.044 |
|  |  | (0.072) | (0.073) | (0.067) |  | (0.059) | (0.061) | (0.052) |
|  |  | ** | ** | ** |  |  |  |  |
| Age |  | -0.137 | -0.134 | -0.125 |  | 0.014 | 0.025 | 0.032 |
|  |  | (0.028) | (0.029) | (0.026) |  | (0.038) | (0.040) | (0.037) |
|  |  | *** | *** | *** |  |  |  |  |
| Education Level |  | -0.089 | -0.086 | -0.072 |  | -0.126 | -0.110 | -0.098 |
|  |  | (0.047) | (0.047) | (0.048) |  | (0.070) | (0.070) | (0.062) |
| Length of watching |  | 0.003 | -0.003 | -0.005 |  | -0.035 | -0.038 | -0.035 |
|  |  | (0.022) | (0.022) | (0.022) |  | (0.036) | (0.032) | (0.030) |
| *Broadcaster level* | | | | | | | | |
| ID |  | -0.440 | 0.308 | 2.029 |  | 0.118 | -0.495 | -0.056 |
|  |  | (1.002) | (0.320) | (0.504) |  | (1.368) | (0.391) | (0.944) |
|  |  |  |  | *** |  |  |  |  |
| BCL |  | -0.423 |  |  |  | -0.007 |  |  |
|  |  | (1.014) |  |  |  | (1.222) |  |  |
| BAL |  |  | -0.300 |  |  |  | -1.066 |  |
|  |  |  | (0.468) |  |  |  | (0.473) |  |
|  |  |  |  |  |  |  | * |  |
| BSL |  |  |  | 1.854 |  |  |  | 0.186 |
|  |  |  |  | (0.507) |  |  |  | (0.909) |
|  |  |  |  | *** |  |  |  |  |
| BCL*ID |  | 0.236 |  |  |  | 0.049 |  |  |
|  |  | (0.259) |  |  |  | (0.350) |  |  |
| BAL*ID |  |  | 0.070 |  |  |  | 0.296 |  |
|  |  |  | (0.114) |  |  |  | (0.129) |  |
|  |  |  |  |  |  |  | * |  |
| BSL*ID |  |  |  | -0.404 |  |  |  | 0.091 |
|  |  |  |  | (0.130) |  |  |  | (0.243) |
|  |  |  |  | ** |  |  |  |  |
| Within-team variance σ^2^ | 0.325 | 0.244 | 0.247 | 0.243 | 0.356 | 0.296 | 0.292 | 0.290 |
| Between-team variance τ_00_ | 0.013 | 0.614 | 0.299 | 0.311 | 0.000 | 1.858 | 1.622 | 1.012 |
| Deviance | 458.681 | 403.484 | 412.141 | 400.982 | 475.291 | 468.383 | 470.310 | 460.277 |

*p≤.05.

**p≤.01.

***p≤.001.

**Table 7.** The HLM results of broadcaster leadership, ITD and loyalty

|  | CGL | | | | CAL | | | |
| --- | --- | --- | --- | --- | --- | --- | --- | --- |
| Dependent variable | Null | M5 | M7 | M9 | Null | M14 | M16 | M18 |
| *Audience level* | | | | | | | | |
| Intercept | 4.125 | 1.339 | 4.293 | 2.181 | 3.642 | 8.569 | 5.559 | 0.912 |
|  | (0.043) | (3.177) | (1.349) | (3.262) | (0.034) | (3.740) | (1.182) | (2.939) |
|  | *** |  | ** |  | *** | * | *** |  |
| Gender |  | 0.125 | 0.172 | 0.188 |  | 0.030 | -0.012 | 0.008 |
|  |  | (0.060) | (0.059) | (0.055) |  | (0.052) | (0.054) | (0.047) |
|  |  | * | ** | ** |  |  |  |  |
| Age |  | -0.125 | -0.120 | -0.116 |  | 0.019 | 0.023 | 0.025 |
|  |  | (0.028) | (0.029) | (0.028) |  | (0.033) | (0.035) | (0.035) |
|  |  | *** | *** | *** |  |  |  |  |
| Education Level |  | -0.060 | -0.060 | -0.051 |  | -0.082 | -0.090 | -0.078 |
|  |  | (0.041) | (0.042) | (0.042) |  | (0.063) | (0.064) | (0.060) |
| Length of watching |  | 0.025 | 0.017 | 0.017 |  | 0.000 | -0.018 | -0.010 |
|  |  | (0.019) | (0.018) | (0.018) |  | (0.030) | (0.029) | (0.028) |
| *Broadcaster level* | | | | | | | | |
| ITD |  | 0.305 | 0.085 | 0.207 |  | 3.141 | -0.446 | 0.263 |
|  |  | (0.828) | (0.351) | (0.834) |  | (1.046) | (0.353) | (0.756) |
|  |  |  |  |  |  | ** |  |  |
| BCL |  | 0.400 |  |  |  | 2.832 |  |  |
|  |  | (0.818) |  |  |  | (0.974) |  |  |
|  |  |  |  |  |  | ** |  |  |
| BAL |  |  | -0.546 |  |  |  | -1.105 |  |
|  |  |  | (0.456) |  |  |  | (0.419) |  |
|  |  |  |  |  |  |  | ** |  |
| BSL |  |  |  | 0.162 |  |  |  | 0.414 |
|  |  |  |  | (0.863) |  |  |  | (1.245) |
| BCL*ITD |  | 0.037 |  |  |  | -0.709 |  |  |
|  |  | (0.214) |  |  |  | (0.271) |  |  |
|  |  |  |  |  |  | * |  |  |
| BAL*ITD |  |  | 0.134 |  |  |  | 0.297 |  |
|  |  |  | (0.114) |  |  |  | (0.118) |  |
|  |  |  |  |  |  |  | * |  |
| BSL*ITD |  |  |  | 0.062 |  |  |  | 0.026 |
|  |  |  |  | (0.220) |  |  |  | (0.200) |
| Within-team variance σ^2^ | 0.325 | 0.241 | 0.244 | 0.244 | 0.356 | 0.288 | 0.287 | 0.283 |
| Between-team variance τ_00_ | 0.013 | 0.614 | 0.284 | 0.420 | 0.000 | 0.108 | 0.093 | 0.057 |
| Deviance | 458.681 | 402.884 | 413.693 | 405.940 | 475.291 | 456.999 | 462.807 | 452.181 |

*p≤.05.

**p≤.01.

***p≤.001.
